# Atypical Presentations of Myocardial Infarction: A Systematic Review of Case Reports

**DOI:** 10.7759/cureus.35492

**Published:** 2023-02-26

**Authors:** Imran A Khan, Habib Md R Karim, Chinmaya K Panda, Ghazal Ahmed, Swatishree Nayak

**Affiliations:** 1 Community and Family Medicine, Baba Raghav Das Medical College, Gorakhpur, IND; 2 Anesthesiology, Critical Care and Pain Medicine, All India Institute of Medical Sciences, Raipur, Raipur, IND; 3 Dermatology, Venereology and Leprosy, All India Institute of Medical Sciences, Deoghar, Deoghar, IND; 4 Ophthalmology, Chandulal Chandrakar Memorial Government Medical College, Durg, IND

**Keywords:** emergency medical service, primary care medicine, acute ischemic heart disease, acute myocardial infarction, clinical features of myocardial ischemia, atypical presentation of myocardial ischemia, acute coronary syndrome

## Abstract

There is a rising incidence of coronary artery diseases and myocardial infarction (MI). Mortality associated with acute MI (AMI) is directly linked to the time to receive treatment and missed diagnoses. Although health professionals are aware of typical AMI presentation, atypical MI is difficult to diagnose, which on the other hand, is likely to have an impact on morbidity and mortality. Therefore, it is prudent to know such atypical presentations, especially for emergency and primary care physicians. We aimed to systematically evaluate the clinical presentations of atypical MI and analyze them to characterize the common clinical presentations of atypical MI. We researched the PubMed database, did citation tracking, and performed Google Scholar advanced search to find the cases reported on the atypical presentation of MI published from January 2000 to September 2022. Articles of all languages were included; Google Translate was used to translate articles published in languages other than English. A total of 496 (56 PubMed articles, 340 citations from included PubMed articles, and 100 articles from Google Scholar advanced search) were screened; 52 case reports were evaluated, and their data were analyzed. Atypical presentations of myocardial infarction are vast; patients may have chest pain without typical characteristics of angina pain or may not have chest pain. No typical characterization could be done. Most patients were in their fifth decade or above of their life and commonly presented with pain and discomfort in the abdomen, head, and neck regions. Prodromal symptoms were consistent findings, and many patients had two to three comorbidities out of four common comorbidities, i.e., diabetes, hypertension, dyslipidemia, and substance abuse. A patient who is 50 years old or more, having comorbidities such as diabetes, hypertension, dyslipidemia, history of tobacco or marijuana usage, presenting with prodromal symptoms like shortness of breath, dizziness, fatigue, syncope, gastrointestinal discomfort or head/neck pain should be suspected for atypical MI.

## Introduction and background

Acute coronary syndrome (ACS) leading to myocardial infarction (MI) is the leading cause of mortality around the globe [1]. Acute MI (AMI) frequently leads to cardiogenic shock. Morbidity and mortality associated with MI are proportionate to the time taken to receive treatment from the onset of symptoms. Studies indicate chest pain as the most common presenting symptom (prevalence nearly 92%) in patients with suspected ACS [2,3]. This chest pain is often described with a few characteristics; retrosternal chest pain of squeezing/tightness/heaviness nature, which gets provoked by exertion or stress and relieved by rest or nitroglycerin. Pain may radiate to the left jaw and shoulder/arm. However, atypical clinical features, including but not limited to neck pain, pain in the back, throat pain, ear discomfort, and hiccups, are not uncommon [4,5]. Craniofacial pain can be the sole symptom in up to 6% of patients with AMI [6]. Women need special mention as they often present with atypical symptoms, and a high index of suspicion is required [7]. It can lead to delayed diagnosis, suboptimal treatment, and detrimental outcomes [8].

Moreover, around 5% of patients with AMI may develop cardiogenic shock with a mortality of 40-50% [9]. To avoid such mishaps, accurate and timely interpretation of atypical clinical symptomatology of AMI has a vital bearing on patient triage, treatment, and subsequent management. For example, improved survival has been demonstrated by the timely implementation of suitable reperfusion therapy [10]. With this background, we conducted a literature search and review related to atypical presentations of MI so that patients with such presentations can be suspected easily. Further, we also aimed to characterize the common atypical presentation, if possible.

## Review

Study design and selection

The present unregistered systematic review was conducted by searching the medical database in PubMed using PubMed advanced search engine and Medical Subject Heading (MeSH) index term combination of ("myocardial infarction"[Title/Abstract]) AND ("atypical presentation"[Title/Abstract]) to find the relevant articles published between 01/01/2000 and 30/09/2022. Further, we did a citation tracking of the included articles from the PubMed search process to collect and synthesize different atypical presentations of myocardial ischemia mentioned in recent literature. Furthermore, Google Scholar advanced search in the article's title with the words "myocardial infarction" (with all the words) and "atypical" (with at least one of the words) fields of the search engine was done. The period for the Google Scholar search was also limited to the same period as for PubMed. The article was searched by two researchers (Habib Md R. Karim [HK] and Ghazal Ahmed [GA]). Although our study does not require meta-analysis, the search methodology and result reporting are done as per the Preferred Reporting Items for Systematic Reviews and Meta-Analysis (PRISMA) guidelines for the applicable segments.

Eligibility criteria

We included all case reports, letters, and commentary, with case details for atypical myocardial infarction. If case series reported individual cases, it was included, otherwise excluded. All other types of articles were excluded. If any article was available other than in the English language, we used Google Translate online to read the abstract and determine the suitability of inclusion. We did not include any grey material in the analysis. It was followed by a screening of the article by two researchers (Imran A. Khan [IK] and Chinmaya K. Panda [CP]) who were not involved in the respective database search. Tabulation of the included articles was done jointly by HK and IK, and Swatishree Nayak [SN] and CP did verification.

Statistical analysis

The characteristics (ages, gender, comorbidities, symptomatologies) of the case-based reports were further used to generate a master chart in Excell and analyzed to find the common characteristics of atypical presentation of MI. As no randomized study is feasible according to our study hypothesis and no original article has been analyzed, we have not reported any risk for bias.

Results

**Figure 1:**
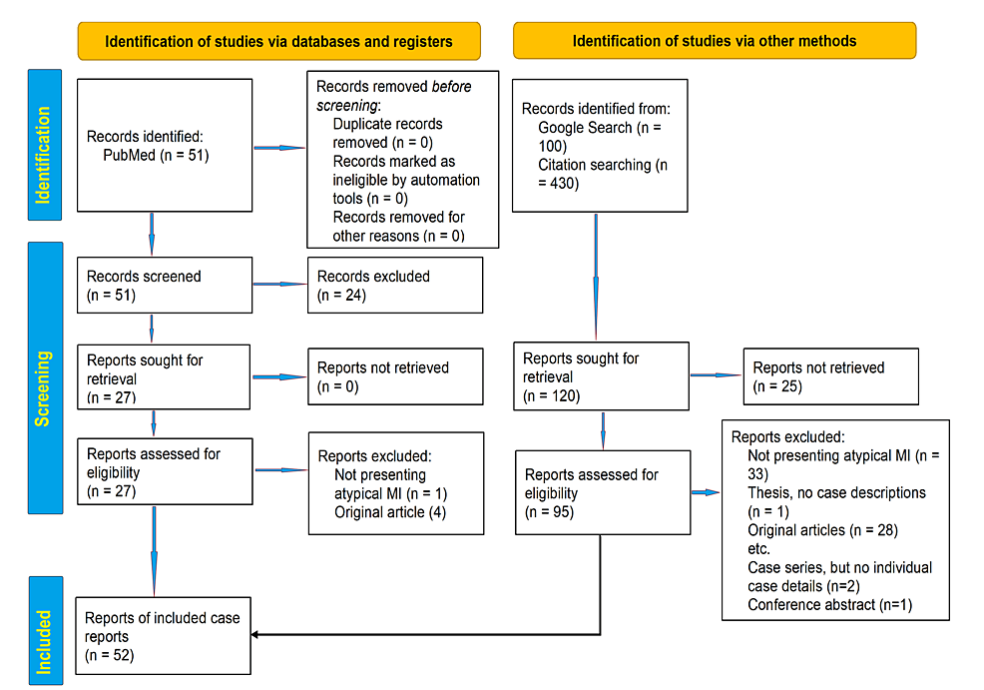
PRISMA 2020 flow diagram MI - myocardial infarction; PRISMA - Preferred Reporting Items for Systematic Reviews and Meta-Analyses

Our PubMed search yielded 51 articles - 26 were related to atypical MI, and 22 were case reports. There were 430 citations in these 26 related articles that were also used for citation tracking, and ten case reports were found eligible for inclusion. Furthermore, the Google Scholar search resulted in 100 articles after filtering the citations, which were screened and analyzed, and another 20 articles were included. The flow diagram depicting the search, screening, and inclusion is presented per the PRISMA 2020 flow diagram in Figure 1 [11].

The characteristics of the included case report-based articles are presented in Table 1.

**Table 1:** Brief description of included articles AF - atrial fibrillation; CAD - coronary artery disease; CRF - chronic renal failure; CVA - cerebrovascular accidents; DM - diabetes mellitus; ESRD - end-stage renal disease; F - female; HTN - hypertension; IHD - ischemic heart disease; M - male, PCI - percutaneous coronary intervention; RBBB - right bundle branch block; TIA - transient ischemic attack; TKR - total knee replacement; TTP - thrombotic thrombocytopenic purpura

No.	Authors (year) [Reference]	N (sex/age in years)	Atypical symptoms	Comorbidities
1	Madias et al. (2002) [12]	Two (M/75, M/62)	Fatigue and lightheadedness, intermittent epigastric discomfort without nausea or vomiting	HTN, RBBB, DM, ESRD, hyperlipemia
2	Timurkaynak et al. (2002) [13]	One (F/28)	Severe, sudden onset of localized back pain and tachypnoea	Smoking, oral contraceptive use, thyrotoxicosis
3	Wittlesey (2002) [14]	One (M/54)	Pleuritic chest pain, worsening with movement and deep breathing, weakness, and fatigue	DM, HTN, Reiter's syndrome
4	Dwyer (2003) [15]	One (F/64)	Mid-back pain, dizziness, persistent cough	Chronic renal infection, RBBB, atrial premature contractions
5	Ivanović et al. (2005) [16]	One (M/44)	Atypical chest pain during exercise	None
6	Zeina et al. (2006) [17]	One (M/23)	Intermittent left-sided atypical chest pain	Hyperlipidemia
7	Wei et al. (2008) [18]	Two (M/36, F/85)	Intolerable and refractory headache, eyeball pain, atypical chest pain	CVA
8	Lin et al. (2009) [19]	One (M/83)	Severe and diffuse bone pain	DM
9	Siau K. (2009) [20]	One (M/61)	Pain along the medial aspect of his left thigh	Recurrent reflux oesophagitis
10	Kohli et al. (2010) [21]	One (F/66)	Sudden onset of slurred speech preceded by dizziness and vomiting	None
11	Amirhaeri et al. (2010) [22]	One (F/53)	Earache and sore throat	Smoker, dyslipidemia, DM, post-PCI
12	Davenport et al. (2012) [23]	One (M/68)	Intractable hiccups causing insomnia	DM, HTN, tobacco use
13	Her et al. (2012) [24]	One (F/54)	Atypical chest pain	Papillary fibroelastoma of the aortic valve
14	Holm et al. (2012) [25]	One (M/48)	Leg pain due to arterial occlusion	Smoking
15	Khoroshikh (2013) [26]	One (M/57)	Atypical hand pain, shortness of breath while walking	HTN, hypercholesterolemia, smoking
16	Abdalla et al. (2014) [27]	One (M/65)	Progressive dyspnoea, presyncope	Morbid obesity, post gastric bypass, depression, alcohol dependence
17	Basic-Jukic et al. (2014) [28]	One (M/55)	Otalgia on post-op renal transplant day three	DM, coronary stent, post-renal transplant
18	Dundar et al. (2014) [29]	One (F/87)	Otalgia with sweating on head and neck	DM
19	Petrović et al. (2014) [30]	One (M/63)	TIA without chest pain, sinus tachycardia	HTN, smoking
20	Parekh et al. (2014) [31]	One (M/55)	Unexplained lethargy and anxiety	CAD, post-stent, and on dual antiplatelet
21	Wang et al. (2014) [32]	One (M/79)	Abdominal pain and fever	Gastric villous adenoma, TTP
22	Ajayi et al. (2015) [33]	One (M/58)	Shortness of breathing, aphasia, with hypoxia	Prostate cancer, HTN, hyperlipidemia, peripheral vascular disease, alcoholism, and past-smoker
23	Taylor (2015) [34]	One (M/44)	Recurrent short-duration atypical chest pain with no radiation and no associated symptoms	Smoking
24	Bharvin et al. (2016) [35]	One (M/34)	Abdominal discomfort, pain, vomiting, and mild breathlessness	Alcohol use
25	Chaturvedi et al. (2016) [36]	One (M/77)	Syncope	HTN, DM
26	Ebrahem et al. (2016) [37]	One (F/39)	Syncopal episodes	Post splenectomy
27	Castillo et al. (2016) [38]	One (F/61)	Right sides arm and scalp numbness	None
28	Ahmed et al. (2017) [39]	One (M/43)	Right otalgia associated with mild dyspnoea and fatigability	DM, dyslipidemia, heavy smoker, family history of HTN and, IHD
29	Anzai et al. (2017) [40]	Three (F/72, M/84, F/89)	Pharyngeal pain/sore throat	Patient 1 - none; patient 2 - CRF, TIA, DM, aortic aneurysm; patient 3 - unstable angina
30	Zhang et al. (2017) [4]	One (M/51)	Intractable hiccups and fever without chest pain	HTN, DM, asthma, and gastritis, marijuana addiction
31	Kyaw et al. (2018) [41]	One (F/74)	Isolated throat pain and Wellens' pattern in the initial EGG	DM, HTN, dyslipidemia, hypothyroidism
32	Ugalde et al. (2018) [42]	One (M/47)	Bilateral otalgia	HTN and CAD
33	Bouthillet et al. (2019) [43]	One (F/75)	Tiredness, nausea, dizziness	HTN, DM, hyperlipidemia, and obesity
34	Broughton et al. (2019) [44]	One (M/59)	Bout of hiccups during hospital admission for septic arthritis	DM
35	Gao et al. (2019) [45]	One (M/46)	Recurrent abdominal pain, constipation, persistent hiccups	Smoker, CVA, cholecystitis
36	Ghazzay et al. (2019) [46]	One (M/55)	Diarrhea, mild lower abdominal pain, and nausea	DM, dyslipidemia, smoking
37	Mehmood et al. (2019) [47]	One (F/25)	Atypical chest pain relieved by deep sternal massage, anxiety	DM
38	Rigueira et al. (2019) [48]	One (F/66)	Atypical chest pain, nausea, and hypersudorese, followed by syncope	Rheumatoid arthritis, HTN, and dyslipidemia
39	Zakaria et al. (2019) [49]	One (F/31)	Epigastric pain, belatedness, and nausea	Post-COVID-19
40	Alahmad et al. (2020) [50]	One (M/35)	Palpitation with De Winter ECG pattern	Palpitation, AF
41	Harikrishnan et al. (2020) [51]	One (M/65)	Fever, cough, shortness of breath	COVID-19
42	Mandal s et al. (2020) [52]	One (F/36)	Back pain in the thoracic region	Polycystic ovarian syndrome
43	Pickard et al. (2020) [53]	One (M/56)	Shortness of breath in the recovery ward after TKR	Follow-up case of TKR
44	Faridi et al. (2021) [54]	One (M/39)	Pain abdomen, palpitation, episodic headache, and shortness of breath	Pheochromocytoma with secondary cardiomyopathy
45	Hovey et al. (2021) [5]	One (M/62)	Persistent hiccups	HTN, DM, CVA
46	Khan et al. (2021) [7]	One (48/F)	Atypical chest pain	None
47	Mayfield et al. (2021) [55]	One (63/F)	Left shoulder pain, tender to touch	Smoking
48	Santana et al. (2021) [56]	One (M/36)	Agitation, auditory hallucination	None
49	Atere et al. (2022) [57]	One (M/58)	Abdominal pain	Myeloproliferative neoplasm
50	Benjanuwattra et al. (2022) [58]	One (M/53)	Haemoptysis	HTN, dyslipidemia
51	Cheema et al. (2022) [59]	One (M/28)	Retrosternal atypical chest pain	Marfan syndrome, post aortic root and arch replacement
52	Kirchner (2022) [60]	One (F/47)	Stressed, jaw pain	None

The included 52 case-based articles presented data from 56 patients; most reported cases were male, i.e., 36 (64.29%). The median, interquartile range of age of the pooled cases was 55.5 (44-65.75) years. The three most common atypical presentations were gastrointestinal discomfort, chest pain without having typical characteristics of angina pectoris, syncope, and cough and breathlessness, while the three most common comorbidities were diabetes, hypertension, and dyslipidemia (Table 2). Prodromal symptoms like dizziness, weakness, and fatiguability were frequently noted in such patients despite not having typical chest pain of acute coronary syndrome (Table 2).

**Table 2:** The derived data and results of common atypical symptoms and comorbidities

Parameters		Values	
Age (in years)		Mean	Standard deviation
		55.14	16.69
Parameters (n=56)		Number	Percentage
Gender	Male	36	64.29
	Female	20	35.61
Common atypical symptoms	Gastri-intestinal discomfort	12	21.43
	Atypical chest pain	10	17.86
	Syncope	7	12.5
	Cough/breathlessness	7	12.5
	Sore throat/throat pain	6	10.71
	Otalgia	5	8.92
	Fatigue	5	8.92
	Dizziness	4	7.14
	Hiccups	4	7.14
	Anxiety	4	7.14
	Fever	4	7.14
	Palpitation	3	5.35
	Mid-back pain	2	3.57
	Headache	2	3.57
Common comorbidities	Diabetes mellitus	17	30.35
	Hypertension	14	25.0
	Dyslipidemia	12	21.43
	Smoking	10	17.87
	Coronary artery disease	7	12.5
	Chronic kidney disease	4	7.14
	Alcohol	3	5.35
	Obesity	2	3.57
	Thyroid abnormality	2	3.57
	COVID-19	2	3.57

Discussion

The present analysis indicates that the atypical presentation of myocardial infarction is wide. We intended to know the atypical presentation as these bear critical value for physicians and the public as patients with AMI having atypical symptoms often delay in care seeking and suspecting or diagnosing by the treating physician resulting in poor prognosis [61]. The prevalence of atypical presentations is high, epidemiological studies indicate that 26% of MI patients can present without typical chest pain [62], and the atypical presentation of MI is nearly 34% [63]. Prodromal symptoms constitute a significant portion of the atypical presentations. Khan et al. [64] analyzed the prodromal symptoms and their gender differences in 1245 patients aged less than or equal to 55 years and found that women had a higher prevalence of prodromal symptoms than men. However, the prodromal symptoms were the same among both gender, i.e., unusual fatigue, sleep disturbances, arm ache/tingling, heart racing (palpitation), frequent indigestion, increased frequency/intensity of headache, etc. Although our analysis of the pooled cases can not indicate the prevalence data, the symptoms, including the prodromal, were similar to those found in the epidemiological studies [62-64]. ACS is a common and potentially life-threatening condition encountered in emergency departments (ED). Clinical symptoms are usually the first step for risk stratification of ED patients presenting with suspected ACS. Despite its dreaded nature, 33% of ACS may mislead clinicians with atypical presentations [65]. The atypical symptoms tend to occur more commonly among older, female, diabetic (possibly due to autonomic neuropathy), hypertensive, and with prior heart failure. They were reported in 5.7% and 12.3% of patients with unstable angina and non-ST elevation myocardial infarction (NSTEMI), respectively [66]. A delay in the diagnosis of ACS has been associated with poor clinical outcomes and increased mortality. Moreover, women have shown increased mortality than men, and increased atypical symptom presentations associated with myocardial infarction in women are thought to contribute to delayed presentation, lower diagnosis and treatment rates might be leading to worse outcomes [67]. International guidelines reinforce the view that atypical symptoms, such as epigastric pain, dyspepsia, or breathlessness, are more likely to present in women [68]. Although our analysis of the reported cases showed a more significant number of cases in males, it cannot refute the female preponderance as it is not an epidemiological study but rather an analysis of the published atypical cases, which represents the tip of the iceberg only.

Somatic referred pain is felt at a distant site other than the noxious stimulus-affected area, often described as dull with poor localization. The pain may radiate to corresponding dermatomes such as the epigastrium, shoulders, arms, back (interscapular region), lower jaw, and neck [69]. There are usually no neurological symptoms or weakness because there is no compression or direct injury of the spinal nerves. The pathophysiology is thought to be related to the neuroanatomy of the nerves innervating the heart and dermatome. Convergence-projection theory has been postulated as a cause of this referred pain. This occurs due to the incapability of the central nervous system to differentiate between stimuli that converge on common sensory pathways [70]. Our analysis showed that myocardial infarction could present with atypical chest pain or, most of the time, without chest pain. Although the common symptoms are gastrointestinal, cough, throat pain, and otalgia were also found in nearly 10% of cases, a few patients even presented with eyeball pain, pain along the medial aspect of their left thigh, left shoulder pain, agitation, auditory hallucination, hemoptysis, severe and diffuse bone pain, leg pain, atypical hand pain, jaw pain, right sides arm, and scalp numbness. All these vast types of pain, which are not even related to the dermatomes of the heart and chest, made it difficult to find a link between them. The same was also noted for associated comorbidities. While hypertension (HTN), diabetes mellitus (DM), dyslipidemia, and smoking have been linked in the epidemiological studies as risk factors [71], several other comorbidities were noted in our pooled cohort, i.e., recurrent reflux oesophagitis, gastric villous adenoma, thrombotic thrombocytopenic purpura, depression, post-surgery, asthma, polycystic ovarian syndrome, Reiter's syndrome, prostate cancer, myeloproliferative neoplasm, rheumatoid arthritis. However, our study can not ascertain their association with atypical presentation owing to the non-epidemiological nature of the methodology and the smaller pooled sample. Although some patients did not have proven risk factors for coronary artery disease (CAD), they had some other cardiac or vascular pathologies like pheochromocytoma with secondary cardiomyopathy, Marfan syndrome, post aortic root and arch replacement, cerebral infarction, peripheral vascular disease, papillary fibroelastoma of the aortic valve. Some patients also had a family history of HTN and ischemic heart disease (IHD) or had personal habits like tobacco chewing and marijuana addiction. Notably, 86% of the patients had comorbidities, and the majority had more than one. Interestingly two out of 57 patients also had post-COVID-19 status without any other comorbidities. While diabetes, HTN, dyslipidemia, and substance abuse are expected comorbidities, the intriguing finding was that nine (15.8%) patients had three, and another eight (14%) patients had two out of these comorbidities. 

Our systematic review has only case reports, which, as such, have a low level of evidence. Although attrition, allocation, selection, blinding, and statistical analysis-related biases are unlikely in our selected articles, selective reporting of the symptoms and publication can still be possible. Further, not all cases are reported in the literature; instead, it is usually noted that unusual or unique cases are only published, so the impact of publication bias is very much feasible. The other limitation of our review is that we have not searched Embase or Web of Sciences as these were not freely accessible, which also have vast data. Although Google Scholar search and citation tracking are likely to cover up the limitation, there is still a possibility that a few cases might have been left out. We also limited our data research, including articles from the year 2000 and afterward. It was done with the intention to keep the data relevant to recent times as the definition of MI, their management, and even reporting of the cases in academic journals have undergone significant changes over the last few decades. 

## Conclusions

This review highlights the atypical presentations of myocardial infarctions. The analysis findings indicate that a primary care or ED physician should suspect atypical presentation of myocardial infarction even in patients who do not have chest pain or have chest pain but not having typical characteristics of angina pain. A patient is in the fifth decade of life or above, having comorbidities like DM, HTN, dyslipidemia, or having a habit of tobacco or marijuana use, significantly if associated with prodromal symptoms like feeling breathlessness, dizziness, fatiguability, or syncope, is likely to present with atypical presentation. As early diagnosis and intervention can save a life in AMI, such patients can be investigated to rule out AMI.
